# Quantifying calcium carbonate and organic carbon content in marine sediments from XRF-scanning spectra with a machine learning approach

**DOI:** 10.1038/s41598-022-25377-x

**Published:** 2022-12-02

**Authors:** An-Sheng Lee, Weng-Si Chao, Sofia Ya Hsuan Liou, Ralf Tiedemann, Bernd Zolitschka, Lester Lembke-Jene

**Affiliations:** 1 Institute of Geography, University of Bremen, Bremen, Germany; 2 Department of Geosciences and Research Center for Future Earth, National Taiwan University, Taipei, Taiwan; 3 Alfred-Wegener-Institut Helmholtz-Zentrum für Polar- und Meeresforschung, Bremerhaven, Germany

**Keywords:** Palaeoceanography, Computer science, Scientific data

## Abstract

Geochemical variations of sedimentary records contain vital information for understanding paleoenvironment and paleoclimate. However, to obtain quantitative data in the laboratory is laborious, which ultimately restricts the temporal and spatial resolution. Quantification based on fast-acquisition and high-resolution provides a potential solution but is restricted to qualitative X-ray fluorescence (XRF) core scanning data. Here, we apply machine learning (ML) to advance the quantification progress and target calcium carbonate (CaCO_3_) and total organic carbon (TOC) for quantification to test the potential of such an XRF-ML approach. Raw XRF spectra are used as input data instead of software-based extraction of elemental intensities to avoid bias and increase information. Our dataset comprises Pacific and Southern Ocean marine sediment cores from high- to mid-latitudes to extend the applicability of quantification models from a site-specific to a multi-regional scale. ML-built models are carefully evaluated with a training set, a test set and a case study. The acquired ML-models provide better results with R^2^ of 0.96 for CaCO_3_ and 0.78 for TOC than conventional methods. In our case study, the ML-performance for TOC is comparably lower but still provides potential for future optimization. Altogether, this study allows to conveniently generate high-resolution bulk chemistry records without losing accuracy.

## Introduction

Over the last decades, we have been experiencing the rapid development of machine learning (ML)^1,2^. It has been employed in different domains and tasks to improve performance by leveraging the collected data. Although ML has been transformative in many fields, there is still a lack of knowledge and applications in traditional science. In this study, we aim to improve this process by introducing ML to quantify and predict oceanographical data from marine sediments.

Marine sediments are prime recorders of Earth’s environmental history through the steady accumulation of biogenic and lithogenic detritus. Qualitative and quantitative analyses of sedimentary components and their chemical properties are essential in providing information for reconstructing paleoclimatic and paleoceanographic changes from annual to orbital, and even tectonic timescales^3^. Calcium carbonate (CaCO_3_) as a principal biogenic component of pelagic marine sediments and acts as a major factor in the oceanic carbon system, which in turn regulates natural atmospheric CO_2_ variations to a large extent^4^. Determination of the CaCO_3_ content in weight percent (wt%) is commonly calculated from the difference between total carbon content and total organic carbon content (TOC; see “Materials and methods”). However, laboratory analyses are time- and labor-consuming, restricting the attainable temporal and spatial resolution.

With the advantages of high-resolution (≥ 100 μm), non-destructive and rapid measurements, X-ray fluorescence (XRF) core scanning techniques may lift the restriction of resolution. An opened 1 m-long marine sediment core can be scanned from 1 to 3 h, depending on scanning settings, to obtain elemental profiles with a 1 cm spatial resolution and related replicates. This method has contributed significantly to systematically recording high-resolution geochemical profiles of sediments. Moreover, its applicability covers all kinds of natural archives, such as soft sediments, speleothems, corals, rocks and tree sections^5^. Despite these advantages, the method suffers of one major disadvantage: it only provides semi-quantitative measurements. The non-linear relation between quantitative and XRF measurements is caused by physical and matrix effects as well as a general lack of control on measurement geometry^6–8^. As a result, quantification of XRF measurements is in high demand.

There are several stepstones on the path to quantifying XRF measurements of sediment cores. Several attempts of quantifying elemental concentrations from the XRF intensity data via direct regression (i.e., Ordinary Squares Regression) have achieved success (e.g.,^7,9,10^). Weltje and Tjallingii^11^ improved the quantification by introducing the additive log-ratio and the major axis regression to the workflow. The relation between elemental concentration and XRF elemental intensities is carefully dealt with by using detailed mathematic derivation (XRF spectrometry theory and statistical theory of compositional data) and empirical tests. However, the power of quantification is limited to “relative” element concentrations, which is constrained by the element assemblage as input. As a follow-up, Weltje et al.^6^ proposed a next level of improvement to increase the accuracy and quantify “absolute” elemental concentrations. They modified the previous workflow^11^ by implementing centred log-ratios and Partial Least Square Regression. The idea of covariance between matrix elements is hence included. Furthermore, it introduces cross-validation (CV) to more rigorously evaluate the model’s predictive power. To make this workflow easier to be applied, the software Xelerate was developed supported by a statistically robust sampling scheme (http://www.mennobloemsma.nl/software.php).

Based on thriving computing power and ML techniques^1,2^, ML applications in the field of geochemical research have been growing (e.g.,^12–14^). Thus, a further stepstone of quantifying XRF measurements can be achieved. First, the application should not be limited to element concentration since there are other proxies, which are of scientific interest and will benefit from high resolution, such as grain size, terrigenous input, opal, CaCO_3_ and organic matter. Second, the elemental intensity to be quantified can be substituted by the emitted fluorescence energy and wavelength spectrum (in short: the XRF spectrum, i.e., the raw data), which records comprehensive information of scanned sediments but is a jump of two orders of magnitude in data dimension. The commonly used elemental intensity is generated by converting the XRF spectrum using data processing software. Fine-tuning of the related software settings needs care and experience. To produce semi-quantitative data rapidly, this procedure often receives less attention than necessary. Consequently, using the XRF spectra avoids this manual bias, especially when dealing with a high quantity of sediment cores. Moreover, most of the XRF scanners cannot determine the lighter elements (atomic number < 11, such as carbon) at the low end of the energy spectrum. ML techniques may overcome this conventional XRF limitation. Third, the logarithmic data space can be extended to an infinite non-linearity space efficiently by a mathematical trick, the kernel function^15^. It is expected to give learning algorithms higher capability to cope with the non-linear relation between semi-quantitative XRF and desired quantitative elemental measurements. Fourth, the application of a model was often considered as site- or even core-specific. Having a larger quantity of data from many cores, a kernelized learning algorithm may lift this limitation and builds a large regional quantification model, which smoothens the process of acquiring high-resolution quantitative data.

**Figure 1:**
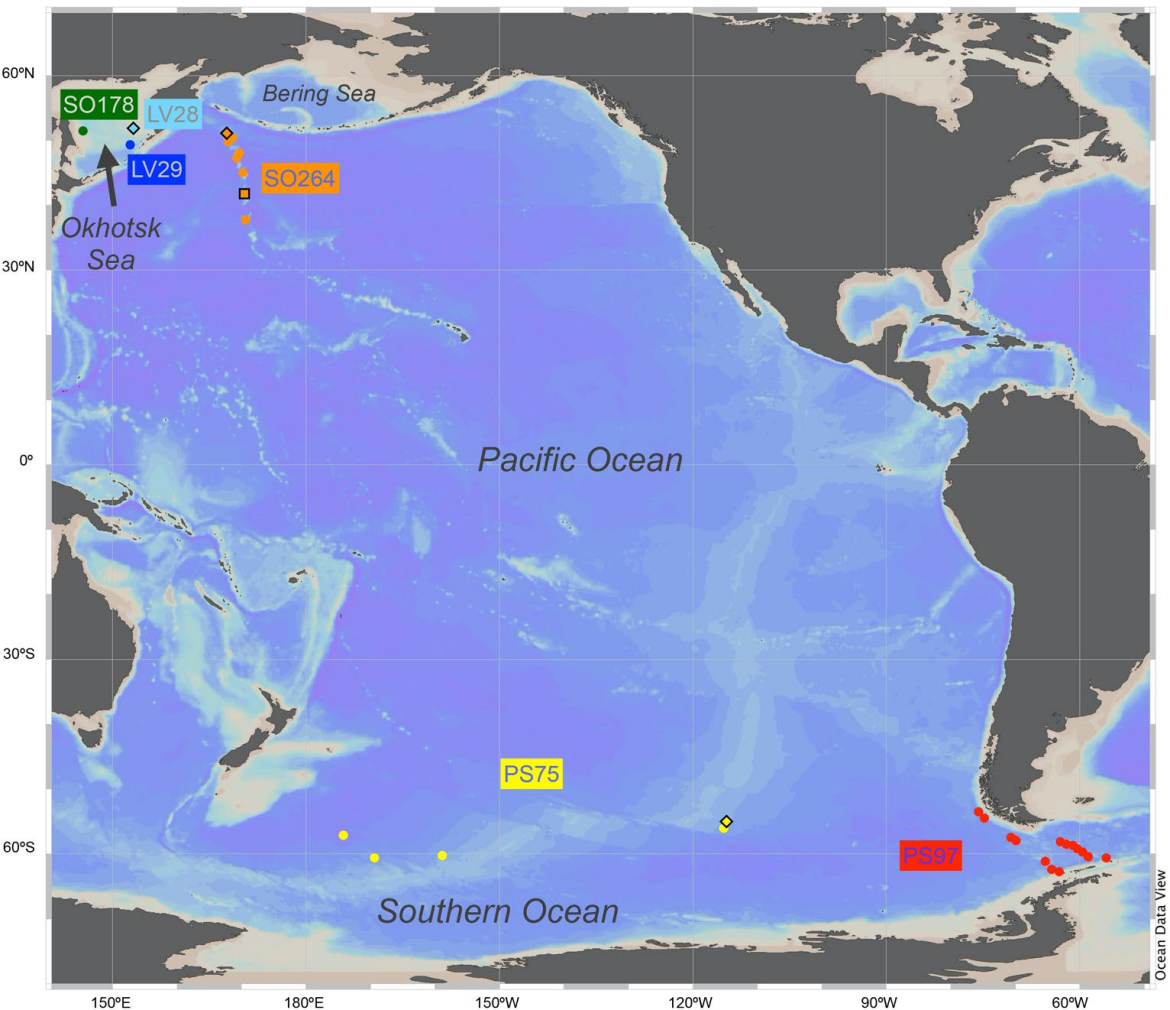
Map of selected pelagic sediment cores, marked by colored dots related to individual and labelled cruises. The orange square represents the pilot test core SO264-15-2. The diamonds represent the cores PS75/056-1, LV28-44-3 and SO264-69-2 used in the case study. Map is created with Ocean Data View 5.6.3 (https://odv.awi.de/).

**Figure 2:**
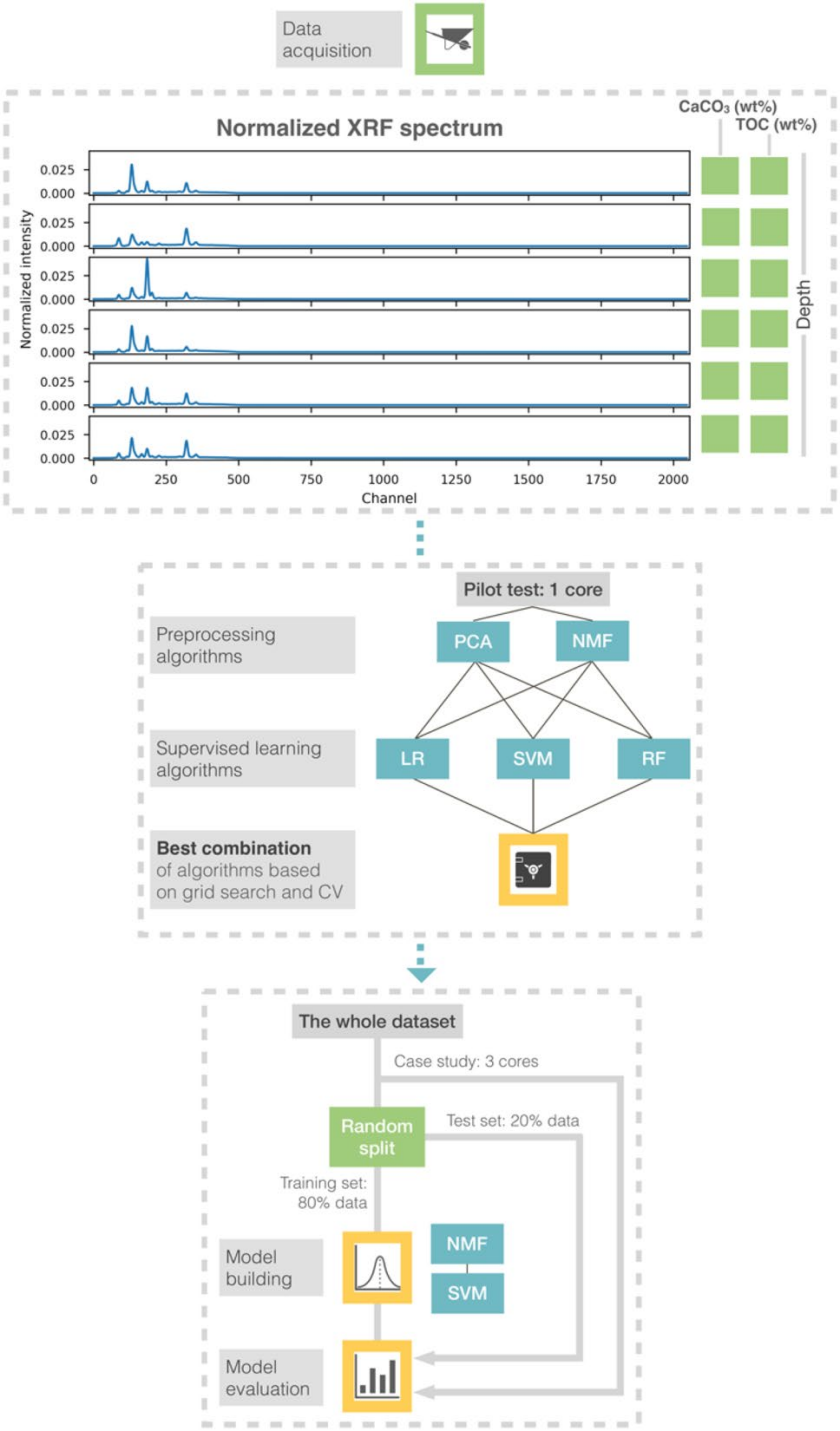
Workflow for the machine learning approach applied to XRF spectra.

In this study, we propose an approach of building models quantifying two commonly used geochemical proxies (CaCO_3_ and TOC contents) from XRF spectra. This involves ML techniques and high-performance computing applied to marine sediment cores that cover multiple regions from the northern and southern pelagic Pacific Ocean (Fig. 1). Two preprocessing algorithms, Principal Component Analysis (PCA) and Non-negative Matrix Factorization (NMF) as well as three supervised ML algorithms, Ridge Linear Regression (LR), kernel Support Vector Machine (SVM) and Random Forest (RF), are included for searching the optimal model. The workflow is schematically shown in Fig. 2. Hopefully, this will kick-off opportunities to include more measurements covering more extensive applications for quantification, such as measurements of different proxies and scanner types. Meanwhile, all the executive codes are open source. The users can easily adopt and modify these codes for their own study and needs, rather than being restricted to a certain software.

## Machine learning: approach and set-up

### Pilot test

Quantifying the XRF spectra to bulk geochemistry is a regression task carried out by ML algorithms to learn the relation between both data. To find a suitable combination for preprocessing and supervised learning algorithms, the XRF spectra and CaCO_3_ content from one recently retrieved core (SO264-15-2) were selected as pilot data. The dataset is composed of paired available geochemistry data and XRF spectra with corresponding core depth (data amount: 40). We tested two preprocessing algorithms (PCA and NMF) and three supervised learning algorithms LR, SVM and RF. PCA and NMF are commonly applied for source separation, extracting vital information from the data^16–18^. SVM has abilities to explore data relations in the infinite non-linear space and tolerate data noise^19^. RF provides a well-regularized learning power in non-linear space based on its tree-based design^20,21^. LR is an L2 regularized Ordinary Least Squares Regression^15^, included as a reference for the performance of the linear algorithm.

The n_component parameter for NMF was initially set following the PCA’s result (explained variances of principal components). The parameters (alpha for LR, C and gamma for SVM, max_depth and n_estimators for RF) control the regularization level of the algorithms, which affect under- and over-fitting issues^22,23^. There is no universally suitable value for these values. Thus, we intuitively grid searched parameters and found a set of optimal parameters, which builds a model with best performance. Since the data is not noise-free, this grid search was integrated with a 5-fold cross-validation (CV)^15^ to be robust. The score (performance) is presented by an averaged coefficient of determination (R^2^) in CV.

### Model training and evaluation

**Figure 3:**
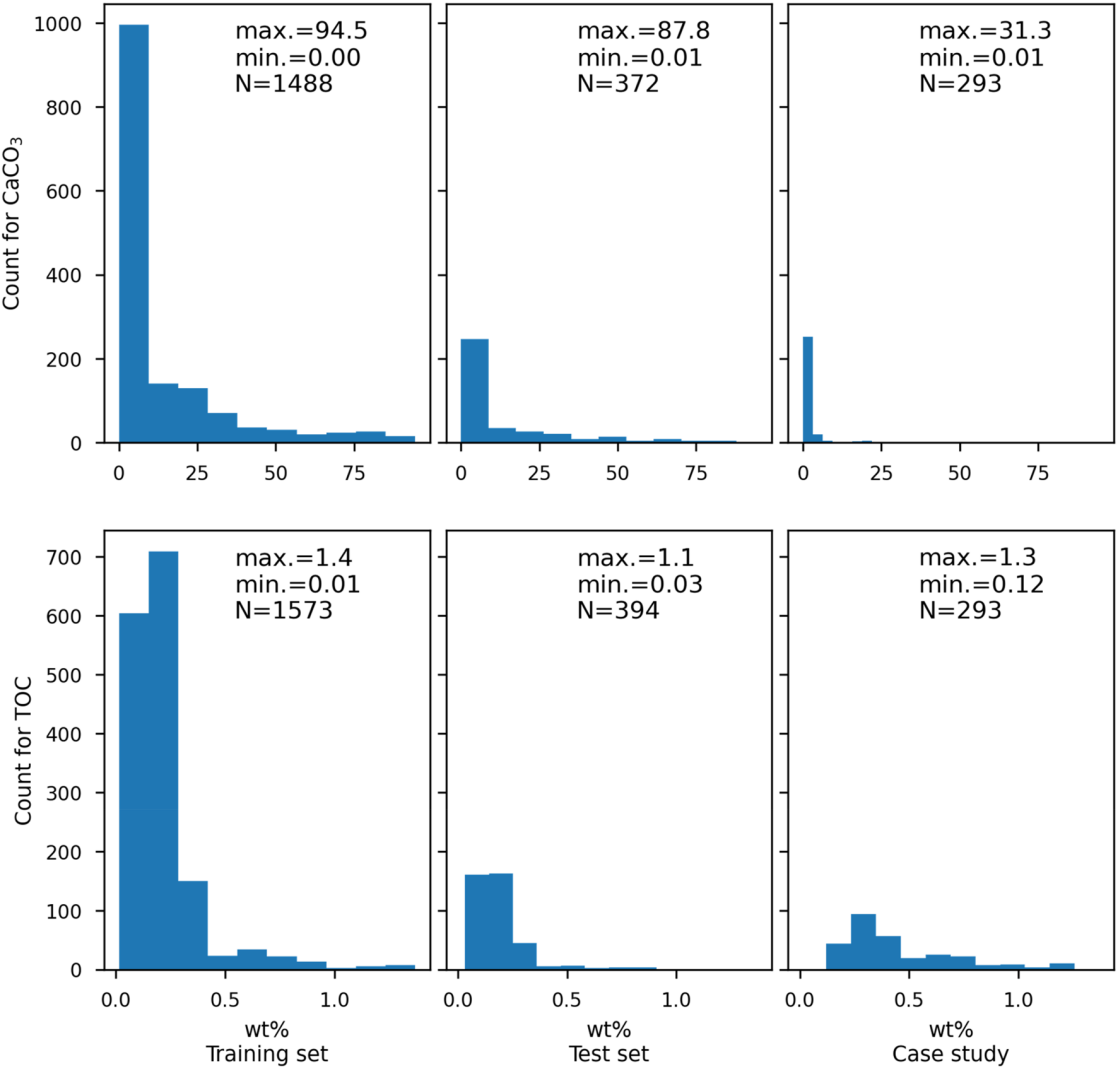
Data distributions of CaCO_3_ and TOC for the training set, test set and case study.

First, the workflow was specified with the best score from the pilot test. Then the training set, which was split from the whole dataset (random 80% of data points, Fig. 3), was used to train our model. The grid search was implemented again for finding optimal parameters, but integrated with 10-fold CV to increase the score’s statistic robustness. The final models for quantifying CaCO_3_ and TOC were subsequently built by adopting the optimal parameters. Details of the grid search strategy are provided by Supplementary Material I.

Evaluating their performance in a pristine data subset is essential to understand the models’ generalization, i.e., how good our models are in quantifying bulk chemistry from other cores. The test set comprises the data left after partitioning the training set from the whole dataset (Fig. 3). Since it is a set of random data, it cannot illustrate a comparison between measurement and prediction for the whole core with uncertainty. In order to rigorously evaluate the models’ performance, we used three additional sediment cores with comparable sedimentary facies applying our optimal models for predictions as a case study (Fig. 1). During evaluation, the models read the XRF spectra of this subset of data to predict CaCO_3_ and TOC contents. The performance was calculated by R^2^, root mean squared error (RMSE) and the ratio of performance to inter-quartile distance (RPIQ)^24^ between actual and predicted values. Meanwhile, errors in the test set and case study were used to construct 95% confidence intervals based on the t-distribution. The uncertainty of our models was thus estimated.

## Results: optimization of machine learning models, evaluations of test set and case study, quantification of CaCO_3_ and TOC

The pilot test shows that the workflow using SVM to learn from the NMF-transformed data provides the most promising results. The linear algorithm (LR) has disadvantages in giving good predictions compared to the SVM workflow. Consequently, the NMF-SVM workflow was carried out for the training set. After grid searching for the parameters, the optimal models were built using the settings listed in Supplementary Material I. The NMF chained in the optimal models of CaCO_3_ and TOC transformed the normalized XRF spectra into fewer features (Figure S4), expected to be vital information and promote the performance of later algorithms.

As conventional methods tend to enhance the accuracy of a model only for training data, they cause a common mistake while evaluating a built model. For example, there are 20 CaCO_3_ and 100 XRF measurements in a core. Operators often try to find a regression between these 20 CaCO_3_ and corresponding XRF measurements giving the highest R^2^. They hence overestimate the regression’s accuracy outside of the training data (20 data points). This is called overfitting in ML tasks^15^. The model’s generalization beyond the training data should be equally important.

Our optimal models were not only evaluated in the training set, estimated in CV scores, but also in the test set and case study, estimated in R^2^, RMSE and RPIQ. Table 1 documents that our models have a good accuracy (CV score and R^2^) for training and test sets, which should exclude the effects of overfitting. The models’ performing statistics in the case study behave differently (Table 1). The performance of the CaCO_3_ model remains moderate, while the one of the TOC model drops significantly compared to its performance in the test set. The 95% confidence intervals estimate constrained uncertainties of the models, which correspond to the statistics in the data subsets. Due to the implementation of the logarithm, our ML approach gives no negative values, which further reduces the prediction’s uncertainty beyond the listed lower confidence interval (Table 1).

**Table 1:** Scores for the optimal models of calcium carbonate (CaCO_3_) and total organic carbon (TOC).

Analyte	Training set CV scores	Test set statistics			Case study statistics			95% CI
		R^2^	RMSE	RPIQ	R^2^	RMSE	RPIQ	
CaCO_3_	0.87	0.96	3.57	4.63	0.61	2.80	0.36	[− 6.77, 5.91]
TOC	0.79	0.78	0.07	1.57	0*	0.37	0.91	[− 0.54, 0.30]

Cross-validation (CV) score stands for the learning performance in the training set, which is the mean of R^2^ during training iterations. R^2^, 95% confidence interval (CI), root mean square error (RMSE) and ratio of performance to inter-quartile distance (RPIQ) estimate the performance and uncertainty of the optimal models in the test set and case study. CI and RMSE both have wt% as the unit. *Negative R^2^ is considered as 0.

**Figure 4:**
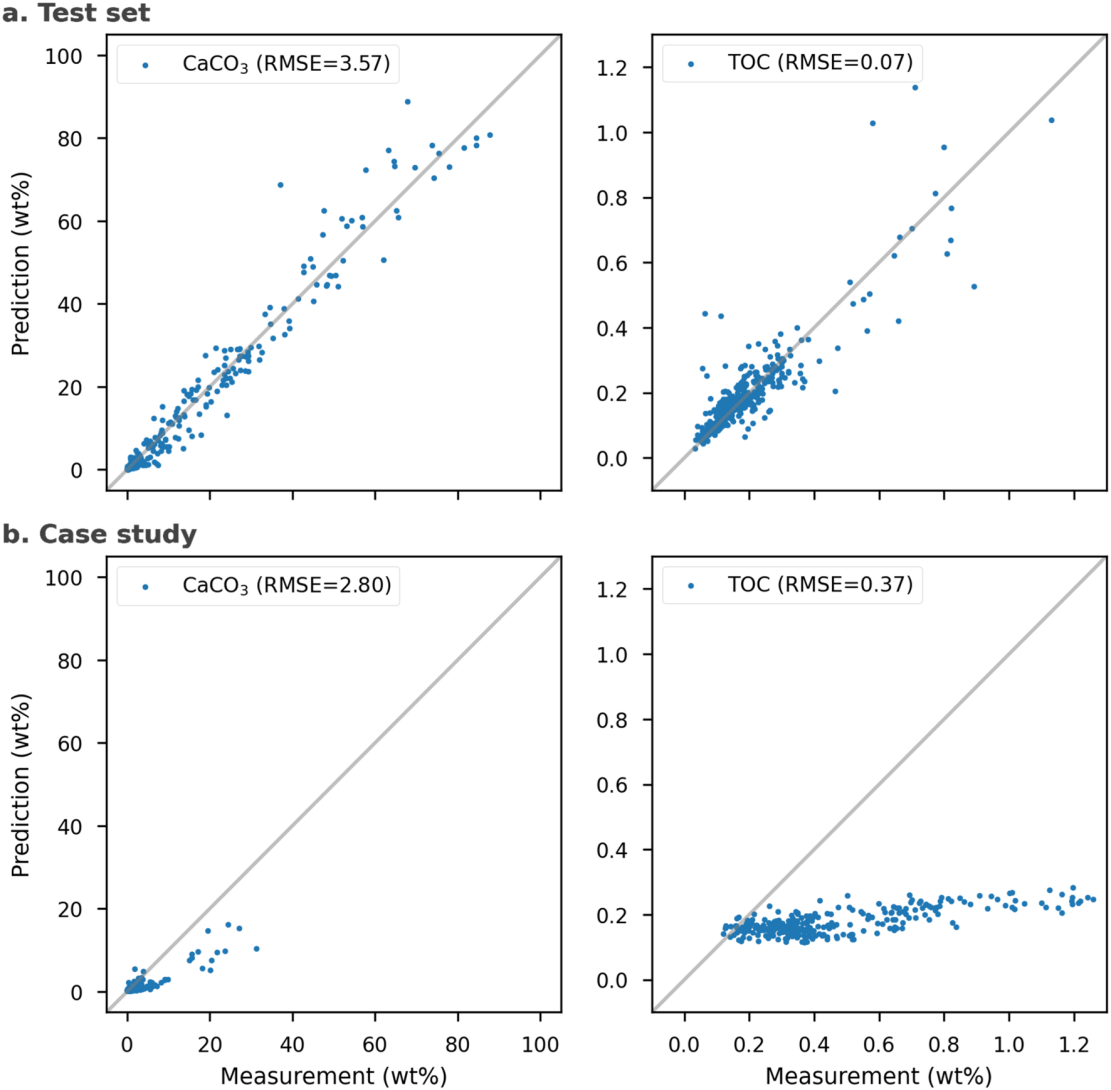
Measured versus predicted CaCO_3_ (left panels) and TOC contents (right panels) in (**a**) the test set and (**b**) the case study.

As shown in Fig. 4a, the error in the test set enlarges when the value increases. This is especially true for TOC but remains acceptable. In Fig. 4b, errors expand when compared to the test set. The values of R^2^ and RPIQ decrease (cf. Table 1). The TOC predictions became worse in R^2^ of two datasets while CaCO_3_ degrades mostly in RPIQ.

The optimal models were implemented to quantify the entire dataset of all cores (i.e., other data without measurements in the same core series of the training and test sets). The total number of data points with bulk geochemistry was increased from < 2000 to 57,240, yielding quantified bulk geochemistry data of CaCO_3_ and TOC for those core intervals, which had not been sampled discretely in high-resolution (1 cm; available in Supplementary Material III). In the quantified data, only 410 data points (0.72%) yield values > 100 wt% and no negative values occurred. Figure 6 illustrates an example for core SO264-55-1 of improved bulk chemistry resolution and surpassing accuracies comparing to the conventional method (XRF-derived proxies).

## Discussion: applications in case study and whole dataset, limitations, strengths

**Figure 5:**
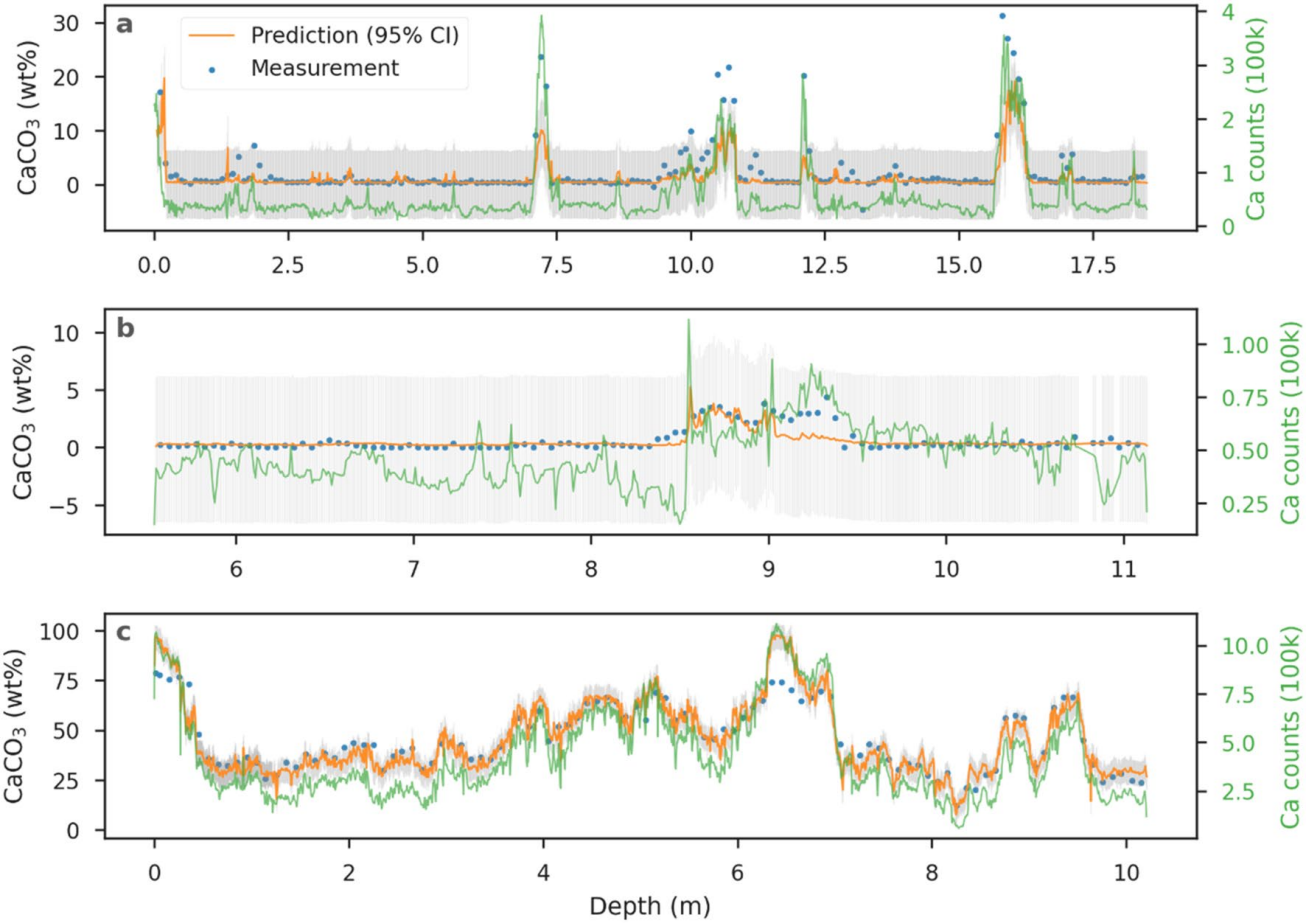
Measured (blue dots) and predicted (orange lines with 95% confident intervals in gray) CaCO_3_ contents compared with the commonly applied XRF-derived proxy (Ca counts as green lines) for cores of the case study (**a**: SO264-69-2, **b**: LV28-44-3, **c**: PS75/056-1).

Since the case study used continuous data from one core, the predictions can be displayed vs depth with both the actual measurements and commonly used XRF-derived element data (e.g., software-processed Ca counts, as the conventional method). The goodness of fit for the CaCO_3_ model (Table 1) is supported by the close correspondence between actual measurements and predictions (Fig. 5). Some extreme values have larger errors in prediction but the performance for moderate values is good. In contrast, the XRF-derived Ca counts show a more unrestrained behavior, in line with some extreme values, but loose accuracy in a general pattern. Thus, the improvement with our ML approach compared to the XRF-derived Ca counts for CaCO_3_ predictions is affirmed.

Overall, the CaCO_3_ prediction in the case study is comparable to the test set (Fig. 4, Table 1), except for RPIQ. The R^2^ degraded more than the RMSE, which is due to a smaller variance in the case study compared to the test set. Discussing RMSE is more adequate in this situation since it is a comparison within the same analyte and value scale (0–100 wt%). The normalization in the calculation of R^2^ is redundant and biased. As a result, the comparable RMSE and almost unbiased error distribution (Fig. 4) guarantee a generalization of the CaCO_3_ model. In other words, this approach provides the opportunity to reduce the CaCO_3_ content measuring time by applying the model to sediment cores. The drop of RPIQ implies that the skewed distribution in the case study may affect the used metrics, which will require more detailed investigation when including more data. Instead of building quantification models core by core, this approach builds a model for each analyte, quantifying cores in a batch process to increase the involved efficiency. The CaCO_3_ model not only shortens the measurement time and the laboratory labor inherent to discrete samples, but also provides an expansion for the use of XRF-scanning spectra.

**Figure 6:**
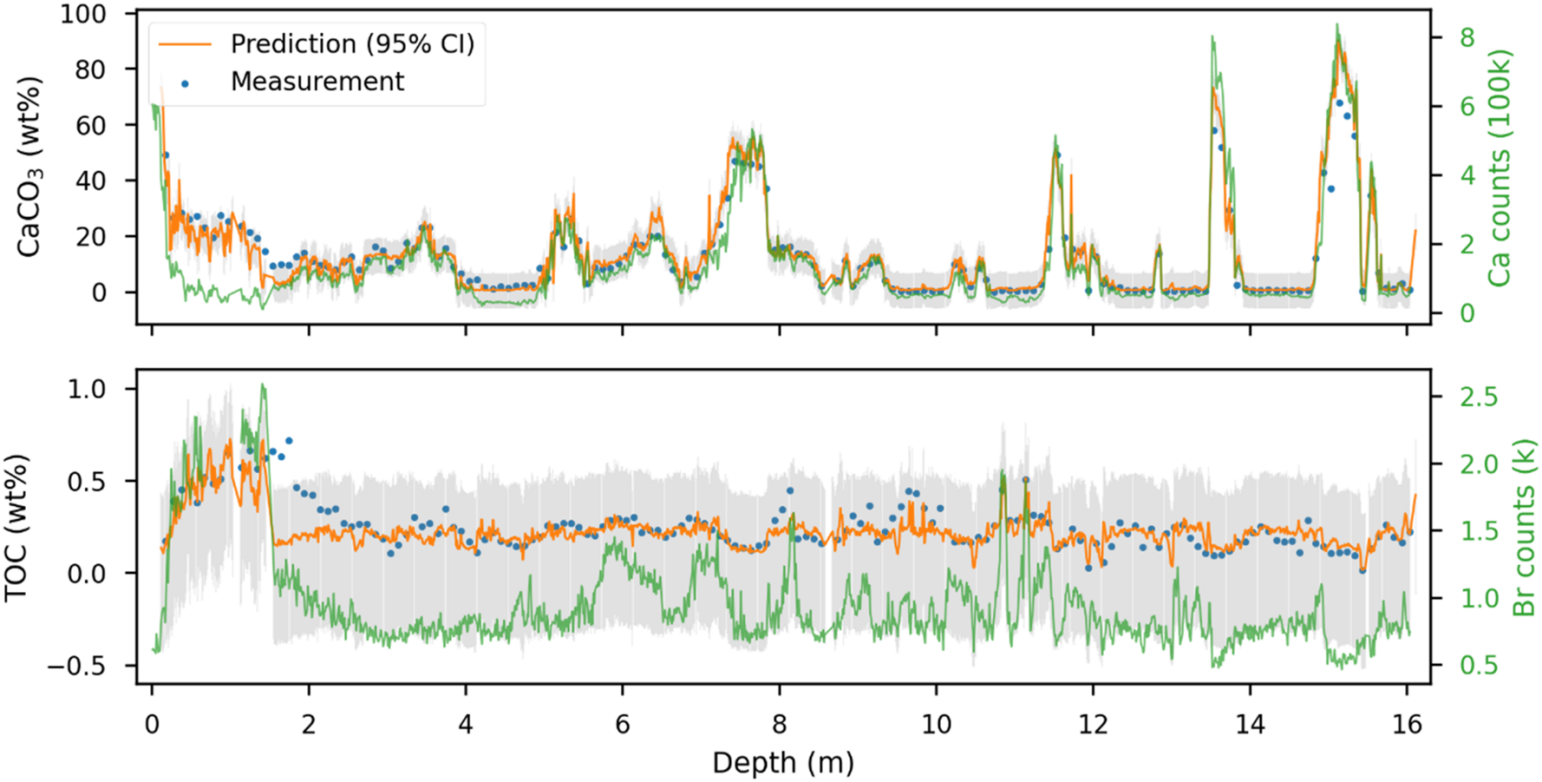
Measured (blue dots) and predicted (orange lines with 95% confident intervals in gray) values for CaCO_3_ and TOC contents in a core from the training set (SO264-55-1). The commonly applied XRF-derived proxies (Ca and Br counts) are marked as green lines.

The TOC performance in the case study was relatively low, compared to that in the test set, indicating the limitation of the generalization of our TOC model. As the debut of studying TOC content directly from XRF spectra, it is an attempt to predict TOC from indirect elemental properties, while carbon itself is beyond the limits of what the scanning detector can measure. Therefore, Bromine (Br) is a commonly applied XRF-derived proxy to estimate marine organic matter (TOC) content (e.g.,^25–27^). But for the case study, Br has a poor fit to the TOC content (Figure S5, Supplementary Material II). We consider that our TOC model develops the regression by collecting the behavior of many different elements falling into the scanner’s capability. The model is learning from indirect and concealed information instead of directly from “visible” signals of organic matter. The correlation between this indirect information and TOC varies with the environmental setting, such as the source of organic matter as supported by the relatively poor fit of the XRF-derived Br from the scanning setting of 30 kV. Thus, the generalization to the cores outside of our initial dataset becomes challenging. Although the generalization of the TOC model is weak to cores beyond our initial dataset, it fulfills our fundamental expectation providing better quantification and higher resolution for our dataset compared to conventional methods (e.g., Fig. 6). The performance in training and test sets gives a R^2^ of 0.78 and 0.79 (Table 1). We assume that applying a full sets of energy spectra, this XRF-ML approach could lead to promising results and predictions of relevant elements and proxies.

The evaluation between test set and case study marks a concealed data-snooping issue. The test set, consisting of the data from the same core series with the training set, overestimates the performance of the TOC model. Hence, our separation of data points between training and test sets is insufficient. The splitting process requires a stricter separation by considering core series. Based on our results, data from the same core is suggested to be split into the same subset (e.g., k-fold splitting iteration with non-overlapping groups^22^). This more rigorous procedure can be found in speech recognition applications that keep voice data from the same speaker in the same data split, even though the content of the voice record is different^23^.

Regarding the very good performance of our CaCO_3_ model, two possible explanations emerge: first, the direct use of XRF spectra instead of elemental intensities after software processing helps the ML algorithms to learn the information without any bias caused by manual fine-tuning. This fine-tuning of software settings needs sufficient experience to adjust for sediment-property changes. The change of water content or organic matter would need corresponding adjustments of the settings. However, these adjustments are commonly ignored due to the lack of experience or tedious labor when encountering long records, which leads to biased elemental intensities. This explanation is supported by the phenomenon that the uppermost part of core SO264-55-1 (Fig. 6) has noticeably biased fitting accuracy compared to the rest of the core for XRF-derived Ca, which was software-processed. This interval could indicate sediment facies changes or very soft sediment conditions and requires respective software parameter fine tuning, which was not applied in this case. Both models overcome this issue to have consistent accuracy. Secondly, compared to software-processed elemental intensities or ratios, the spectrum provides more hidden information recorded in the entire fluorescence signal (e.g., water content). As a result, after learning from a certain amount of data points covering the entire variation of sediment properties, our XRF-ML models are able to determine unbiased multi-elemental and matrix information. This capability of non-linearity solves an existing gap between non-linear XRF elemental proxies and linear bulk chemistry measurements.

Unlike conventional quantification methods, which build site- or core-specific models^6,7^, our approach shows that general models can be constructed accurately for quantifying CaCO_3_ on a larger spatial, regional to basin-wide scale. Thus, a potential demand of repeatedly building and testing traditional regression-based models is eliminated. Eventually, our models could be applied to entire core collections from specified cruises, which cover multiple sectors of e.g., the Pacific Ocean, while significantly reducing the demand of conventional laboratory measurements. With the increased provision of quantitative data by a partially automated process, the temporal and spatial resolution of paleoceanographic and paleoclimatic data can be enhanced significantly.

## Guidelines for future applications

For studies retrieving sediment cores in nearby regions: our CaCO_3_ model is ready to use. No laboratory measurement and high-performance computing is necessary. A common PC with the capability of Python coding is sufficient for adopting our models. For studies featuring cores from other study areas, the model is still worth a trial, but a test run is recommended to evaluate the accuracy. If the model fails or XRF data is acquired from different core scanners, our workflow for building models is well suited for developing own models. For improvement of this first test set of models, such as for future TOC predictions, one should consider using spectra acquired from ideally suited scanner settings, while making sure data from the same core stays in the same subset during the data splitting process. Failure of our model can be related to sediment type, sediment age range, XRF core scanner, X-ray tube and scanning settings. Any major changes of these categories from our dataset might result in notably different XRF spectra and lead to erroneous predictions. The remaining boundary conditions of our dataset are stated in the “Materials and methods” section and ready for future applications.

## Conclusions

A new approach is presented by using machine learning (ML) techniques to build models that can quantify CaCO_3_ and TOC contents directly from XRF-scanning-derived spectra. The advantages of the XRF-ML approach are a quick, quantitative, precise and high-resolution data acquisition, while multiple cores can be calculated simultaneously. The use of XRF spectra reduces manual user-generated bias and increases the input information for ML algorithms. The broad data coverage, the power of ML and computing techniques lift the model’s limitation off the site-specific scale. This novel quantification of CaCO_3_ and TOC contents and the generalization of optimal models is carefully evaluated in training and test sets as well as a case study. The uncertainty of predictions is estimated in 95% confidence intervals from the test set and the case study.

Further cores retrieved from the extratropical North and South Pacific could be quantified with high-resolution (1 cm), hitherto unattainable due to the high amount of analytical workload involved for discrete sample preparation. We provide guidelines for future applications. To progress our multi-region models towards an even broader temporal and spatial domain, we plan to incorporate more measurements, different core locations, scanner types and settings. Hopefully, this approach brings future studies towards retrieving high-resolution quantitative bulk chemistry without losing accuracy.

## Materials and methods

### Sediment cores and bulk measurements

In this study, spectra of 30 XRF-scanned marine sediment cores, including published and unpublished records, together with their corresponding bulk chemistry measurements were examined (in total, 2580 TOC samples and 2517 total carbon content samples; Supplementary Material III). The investigated cores are mostly retrieved from two expeditions: cruise SO264 in the subarctic Northwest Pacific with R/V SONNE in 2018 and cruise PS97 in the central Drake Passage with RV Polarstern in 2016. Another four cores and the case study core were recovered in the Pacific sector of the Southern Ocean during PS75 in 2009/2010. Additional three cores were recovered in the Okhotsk Sea during cruise KOMEX I and KOMEX II with R/V Akademik Lavrentyev in 1998 and cruise SO178 in 2004. The area of all investigated cores is mainly spreading across the high- to mid-latitude Northwest Pacific (37° N–52° N) and the Pacific sector of the Southern Ocean (53° S–63° S), with a water depth coverage from 1211 to 4853 m. The three cores of the case study are located in the Okhotsk Sea (LV28-44-3; 52° 2.5139′ N 153° 5.949′ E; 684 m water depth), the Northwest Pacific (SO264-69-2; 50° 30.877′ N 167° 55.478′ E; 3473 m water depth) and the Southern Ocean (PS75/056-1; 55° 09.74′ S 114° 47.31′ W; 3581 m water depth).

The investigated cores are dominated by pelagic sediments and mainly consist of calcareous and siliceous ooze, and non-carbonaceous fine-grained sediments. We use cores from the pelagic ocean instead of cores close to the shore to avoid influences of coastal erosion and fluvial input (organic matter, terrigenous input). The age of the majority of the sediments ranges from the mid-Pleistocene to the Holocene^28–34^.

To determine bulk sediment parameters immediately after opening of the sediment cores on board, we took syringe samples of 10 cm^3^ in 10 cm intervals from the working halves of the core and transferred them into pre-weighed glass vials. All samples were stored at 4 °C until further shore-based processing. The samples in this study were weighed before and after freeze-drying, homogenized and measured for TC and TOC by a CNS-analyzer (Elementar vario EL III) and a carbon/sulfur analyzer (Eltra CS-800) in the laboratories at AWI in Bremerhaven. The CaCO_3_ content was then calculated from the difference between TC and TOC as follows:

$${\mathrm{CaCO}}_{3}\mathrm{ wt\%}=(\mathrm{TC}-\mathrm{TOC})\times 8.333\times 100\%$$

### Settings of the Avaatech XRF scanning and data compilation

All XRF-scanning measurements were carried out with an Avaatech XRF core scanner at the AWI in Bremerhaven. The X-ray excitation scanning settings were 10 kV at 150 mA with no filter for a count time of 10 s. A rhodium target X-ray tube was deployed. This setting is optimized for producing signals related to CaCO_3_ because it is used as the main estimation for discussing oceanic carbon system. Sample distance was 10 mm, with a 10 mm × 12 mm slit size. In order to include comprehensive information and avoid the software bias, which could be caused by manual operation, the raw spectrum (2048 channels) for each scanning point was chosen instead of processed elemental intensities. The XRF spectra were normalized by the sum of channel signals for each data point to prevent bias from X-ray tube aging. Next, the normalized spectra were standardized to zero mean and unit variance, which balances the importance of each channel number. This is an important pre-treatment prior to PCA and NMF. The bulk geochemistry (CaCO_3,_ TOC) was collected and transformed with a natural logarithm to avoid predicting negative contents later in the model. The XRF spectra were aligned to the bulk chemistry measurements by depth.

### Use of machine learning programming

The developing codes were written in Python scripts and Jupyter notebooks by implementing Python3 built-in functions and SciPy ecosystem packages (Numpy, pandas and scikit-learn)^22,35–39^. The visualizations were conducted using Matplotlib and Seaborn packages^40,41^. We rely on a high-performance computing system managed by Slurm (Klimageographie, University of Bremen) to facilitate the heavy computation for model building. The developing steps were carefully recorded via Git. To promote the FAIR principle^42^, models, codes and package dependencies are all open access on Github (https://github.com/dispink/CaCO3_NWP).

## Supplementary Information

Supplementary Information.

## Data Availability

The datasets generated during and/or analyzed during the current study are available in the Pangaea in accordance with and under the license of CC-BY: Creative Commons Attribution 4.0 International. Chao, Weng-si; Lee, An-Sheng; Tiedemann, Ralf; Lembke-Jene, Lester; Lamy, Frank (2022): XRF down-core scanning and bulk chemistry measurements of sediments from the high latitude sectors of Pacific Ocean. PANGAEA, 10.1594/PANGAEA.949225. The analyzing results is included in the supplement.
