# Supplementary material for: Quantifying calcium carbonate and organic carbon content in marine sediments from XRF-scanning spectra with a machine learning approach
Source: Sci Rep. 2022 Dec 2;12:20860. doi: 10.1038/s41598-022-25377-x (PMC9718834; doi:10.1038/s41598-022-25377-x)
Supplement: Supplementary file 1 — Supplementary Information. [file 41598_2022_25377_MOESM1_ESM.pdf]

Supplementary information for

Quantifying calcium carbonate and organic carbon content in marine sediments from XRF-scanning spectra with a machine learning approach

An-Sheng Lee [1, 2, †] and Weng-Si Chao [*, 3, †], Sofia Ya Hsuan Liou [*, 2], Ralf Tiedemann [3], Bernd Zolitschka [1], Lester Lembke-Jene [3]

[1] University of Bremen, Institute of Geography, Bremen, Germany

[2] National Taiwan University, Department of Geosciences and Research Center for Future Earth, Taipei, Taiwan

[3] Alfred-Wegener-Institut Helmholtz-Zentrum für Polar- und Meeresforschung, Bremerhaven, Germany

[*]Corresponding authors: yhliou@ntu.edu.tw, weng-si.chao@awi.de

[†] These two authors contributed equally to this work.

Contents of this file

I. Detailed grid search results
II. Evaluation in case study
III. Info and exported data

I. Detailed grid search results

During a pilot test, the number of principal components was chosen as 5, i.e. before the explained variance flattened (Figure S 1, Left). This number was also adopted later for the initial grid search of the Non-negative Matrix Factorization (NMF). Figure S 1 (Right) indicates that the combination of NMF and kernel Support Vector Machine (SVM) gives the best performance ($R^2 = 0.91$). The Ridge Linear Regression (LR) shows the slightly worse performance, while the Random Forest (RF) has the worst performance.

There are three parameters that need to be fine-tuned in building models: n_components for NMF, C and gamma for SVM. Based on the Principal Component Analysis (PCA) results of the pilot test (Figure S 1), we initially tried n_components from 5. For each setting of components, ranges of C and gamma have been searched in logarithmic scales to find the model with best performance. When the optimal combination of parameters locates away from the edge of the plot (Figures S 2 and S 3, i.e., ranges of parameters), it is chosen to build the optimal model. The optimal component amount for the TOC model was set to 13, at the edge of the searching range. The reason why upward searching was not continued is that the model's fitting time extends noticeably when increased.

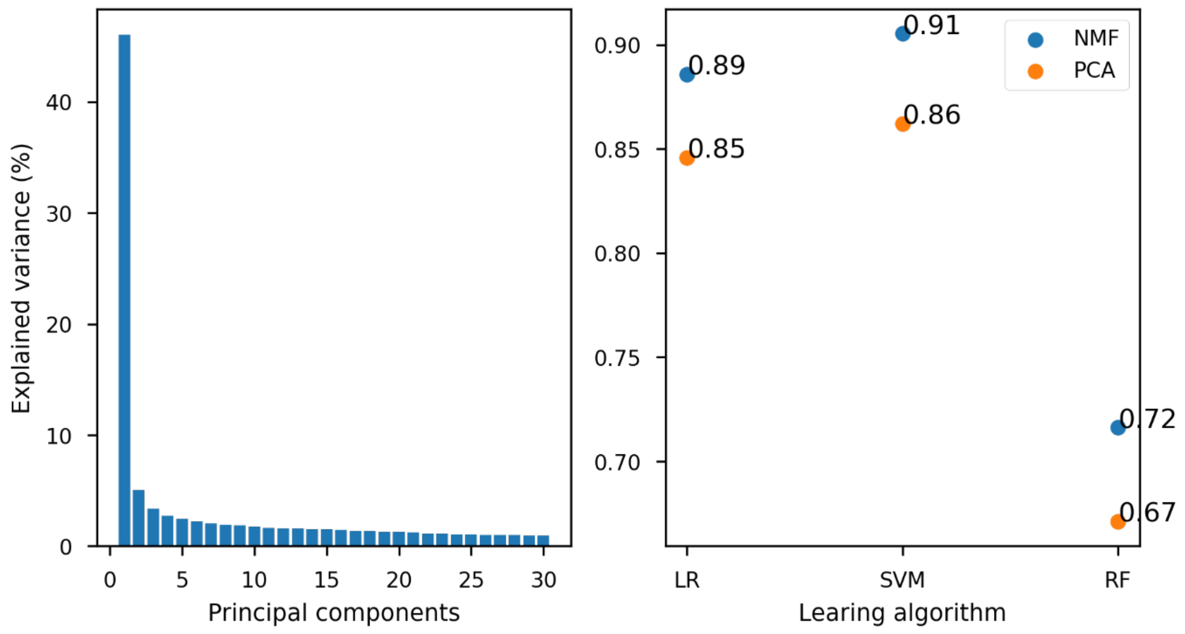

Figure S 1. Results of the pilot test. Left: Explained variance of each principal component. Right: Best performance (R^2) of each workflow.

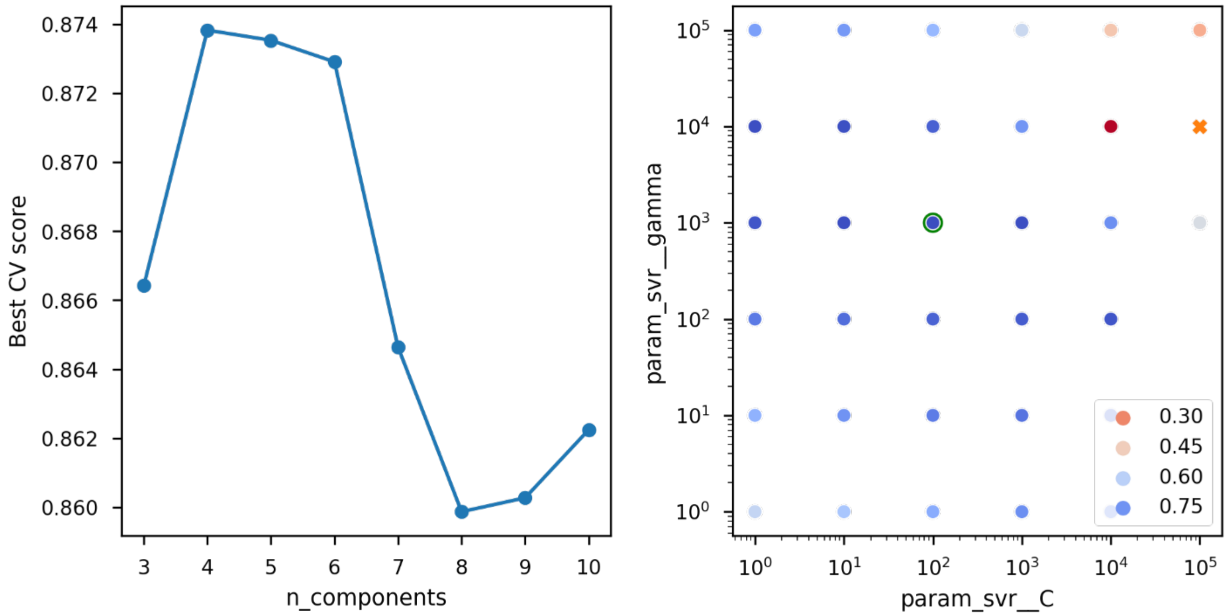

Figure S 2. Visualization of grid search results for $CaCO_3$. Left: Best CV score for each setting of n_components for NMF. Right: CV score grid of the pair of SVM parameters under n_components = 4. The optimal pair of SVM parameters is marked by a green circle.

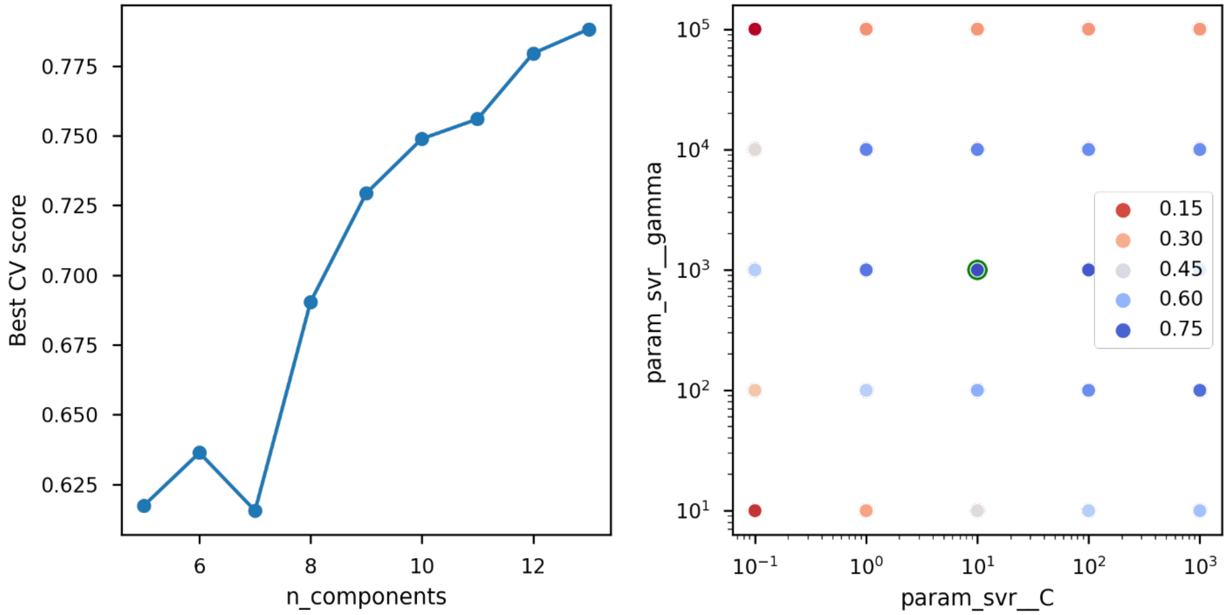

Figure S 3. Visualization of grid search results for TOC. Left: Best CV score in each setting of n_components for NMF. Right: CV score grid of the pair of SVM parameters under n_components = 13. The optimal pair of SVM parameters is marked by a green circle.

Table S 1. Parameter settings for the optimal model for calcium carbonate ($CaCO_3$) and total organic carbon (TOC). n_components is for NMF. C and gamma are for SVM.

Analyte	n_components	C	gamma
$CaCO_3$	4	100	1000
TOC	13	10	1000

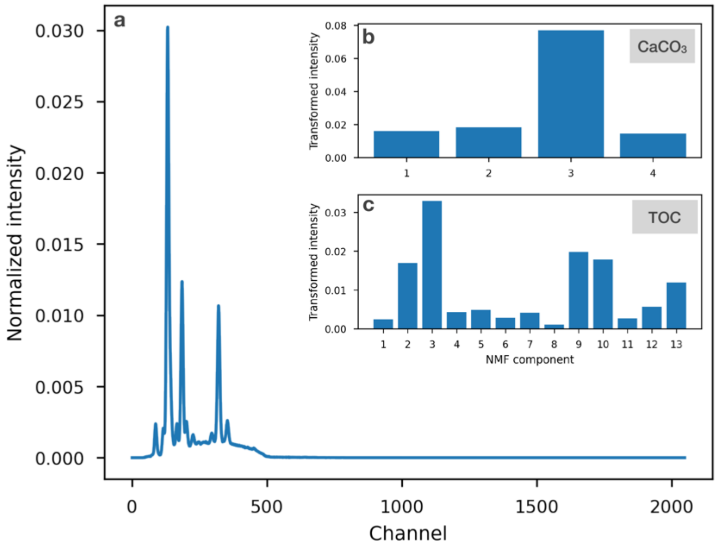

Figure S 4. (a) Example for a normalized spectrum of the data point at 105 mm depth of the case study core (SO264-69-2). Transformed spectra by NMF chained in the optimal models of (b) $CaCO_3$ and (c) TOC from the spectrum (a).

Dataset S1. Grid search results of the pilot research.

pilot_grid_ridge_20210823.csv

pilot_grid_svr_20210318.csv

pilot_grid_rf_20210822.csv

(https://drive.google.com/file/d/1TWIsOJ8UGHBZvvUZ-hiKQpwHGw3Oc4Es/view?usp=sharing)

Dataset S2. Grid search result of building models for $CaCO_3$ and TOC.

grid_caco3+toc_compile_20210823.csv

(https://drive.google.com/file/d/1TWIsOJ8UGHBZvvUZ-hiKQpwHGw3Oc4Es/view?usp=sharing)

II. Evaluation of the case study

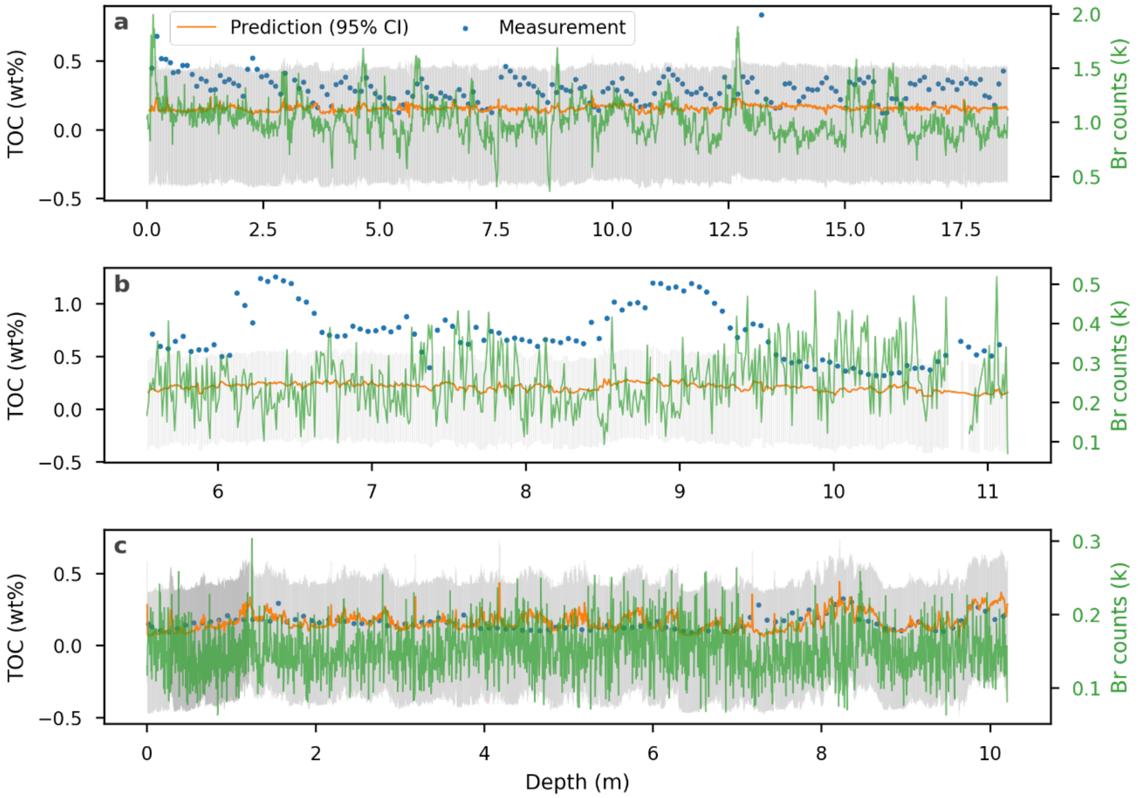

Figure S 5. Measured (blue dots) and predicted (orange lines with 95 % confident intervals as gray bars) TOC contents estimated with the commonly applied XRF proxy Br (counts in green from 30 kV-runs) in the case study cores (a: SO264-69-2, b: LV28-44-3, c: PS75/056-1).

III. Info and exported data

Dataset S3. High-resolution (10 mm) quantified $CaCO_3$ and TOC.

predict_202200629.csv on Pangaea (waiting for Pangaea for data curation)

XRF setting: Avaatech XRF core scanner with the X-ray excitation scanning settings, 10 kV at 150 mA with no filter for a count time of 10 s, and a rhodium target X-ray tube was deployed.

Table S 2. Station list of the studied cores for the whole dataset with relevant information including amount of measurements, dating methods, core age and references.

Station	Latitude (°N)	Longitude (°E)	Water depth (m)	Core length (m)	TOC % data amount	TC % data amount	Bulk measurement reference	Core bottom ca. age (ka)	Chrono-stratigraphic methods	Age data reference	Notes
LV29-114-3	49.375667	152.877933	1762	9.5	72	72	*Max et al. 2012*	26.87 (@5.02m)	^{14}C, XRF*	*Max et al., 2012*	Upper 5 m was studied
SO178-12-3	51.605367	145.4357	1211	16.99	63	n.d.	unpublished data			unpublished data	Upper 6.62 m was studied
SO264-13-2	37.79775	170.72211	3935	13.81	39	39	unpublished data		XRF	unpublished data	
SO264-15-2	41.85472	170.51111	3662	14.48	40	40	*Chao*	947	XRF, Stable isotope	*Chao*	
SO264-28-2	45.00166	170.32305	1935	7.64	39	39	unpublished data	1400	^{14}C, XRF, stable isotope	unpublished data	
SO264-55-1	47.32027	169.4975	2936	16.11	159	159	Chao	450	^{14}C, XRF, stable isotope, tephra	*Chao; Nürnberg, 2018*	
SO264-56-2	47.94222	169.77722	3973	12.66	35	35	unpublished data	1152	XRF, tephra, paleomag.	*Wang et al., 2021; Nürnberg, 2018*	
SO264-64-1	50.12055	168.34666	3495	18.55	36	36	unpublished data	678	XRF	unpublished data	
SO264-66-2	50.26861	168.51138	2751	15.04	40	40	*Chao*	734	XRF	*Chao*	
PS97/27-2	-54.384833	-74.605833	2341.8	2.01	20	20	unpublished data			unpublished data	
PS97/46-4	-60.997167	-65.356167	2775.3	2.26	21	21	unpublished data			unpublished data	XRF only until 126cm
PS97/52-4	-62.499	-64.293667	2890.4	3.14	30	30	unpublished data			unpublished data	
PS97/53-2	-62.662833	-63.0935	2016.1	2	17	17	unpublished data			unpublished data	
PS97/78-1	-60.6525	-55.839667	3666.4	2.23	21	21	unpublished data			unpublished data	
PS97/79-2	-60.142333	-58.991333	3541.3	8.28	83	83	unpublished data			unpublished data	1st core section no XRF
PS97/80-1	-59.674667	-59.631333	3105.9	1.24	11	11	unpublished data			unpublished data	
PS97/83-2	-58.994333	-60.570333	3762.3	6.08	60	60	unpublished data			unpublished data	
PS97/84-1	-58.869	-60.865667	3557	10.94	109	109	unpublished data			unpublished data	1st core section no XRF

PS97/84-1 TC				9	8	8	unpublished data			unpublished data	TC = trigger core
PS97/85-3	-58.3545	-62.167167	3090.8	14.43	145	145	unpublished data			unpublished data	
PS97/85-3 TC				8.9	8	8	unpublished data			unpublished data	TC = trigger core
PS97/89-1	-58.226667	-62.7265	3437.2	10.02	100	100	unpublished data			unpublished data	
PS97/89-1 TC				9	8	8	unpublished data			unpublished data	TC = trigger core
PS97/92-1	-57.763	-69.878833	3823.7	7.89	78	78	unpublished data			unpublished data	
PS97/93-2	-57.499167	-70.274667	3782.2	16.45	162	162	unpublished data			unpublished data	
PS97/128-2	-53.634330	-75.54583	2313.4	10.69	104	104	unpublished data			unpublished data	XRF only until 167cm
PS75/054-1	-56.15175	-115.133033	4113	22.38	224	224	unpublished data	160	XRF	*Benz et al., 2016*	
PS75/083-1	-60.268833	-159.059833	3599	13.13	131	131	unpublished data	268	Fe - dust	*Lamy et al., 2014*	
PS75/083-1 TC				0.37	4	4	unpublished data				TC = trigger core
PS75/093-1	-60.872167	-169.548167	3762	12.84	129	129	unpublished data	528	^{14}C, XRF	*Benz et al., 2016*	
PS75/093-1 TC				0.92	9	9	unpublished data				TC = trigger core
PS75/095-5	-57.019667	-174.43	4853	17.85	177	177	unpublished data				
**Case study core**											
PS75/056-1	-55.162333	-114.7885	3581	10.21	103	103	unpublished data	256	stable isotope	*Ullermann et al., 2016*	
SO264-69-2	50.514480	167.92488	3473	18.55	184	184	unpublished data	450	XRF	*Chao*	
LV28-44-3	52.0419	153.09915	684	11.13	111	111	unpublished data				Upper 5.59 m was studied

* pattern correlation of XRF ratios/elements that is associated between core(s) and published record(s) is used as a chronostratigraphic approach here. See Chao (in prep.) and Max, L et al. (2012).
n.d. = not determined

Reference

Benz, V et al. (2016): Last Glacial Maximum sea surface temperature and sea-ice extent in the Pacific sector of the Southern Ocean. Quaternary Science Reviews, 146, 216-237

Chao, W.-S. et al. Glacial-interglacial variations in productivity and carbonate deposition in the Northwest Pacific during the last 500,000 years. Frontiers in Earth Science (In prep.)

Lamy, F et al. (2014): Increased dust deposition in the Pacific Southern Ocean during glacial periods. Science, 343(6169), 403-407
Max, L et al. (2012): Sea surface temperature variability and sea-ice extent in the subarctic northwest Pacific during the past 15,000 years. Paleoceanography, 27(3), PA3213
Nürnberg, D ed. (2018) RV SONNE Fahrtbericht / Cruise Report SO264 - SONNE-EMPEROR: The Plio/Pleistocene to Holocene development of the pelagic North Pacific from surface to depth – assessing its role for the global carbon budget and Earth ́s climate, Suva (Fiji) – Yokohama (Japan), 30.6. – 24.8.2018.
Ullermann, J et al. (2016): Pacific-Atlantic Circumpolar Deep Water coupling during the last 500 ka. Paleoceanography, 31(6), 639-65
Wang, W et al. (2021). Dating North Pacific Abyssal Sediments by Geomagnetic Paleointensity: Implications of Magnetization Carriers, Plio-Pleistocene Climate Change, and Benthic Redox Conditions. Frontiers in Earth Science, 577.
